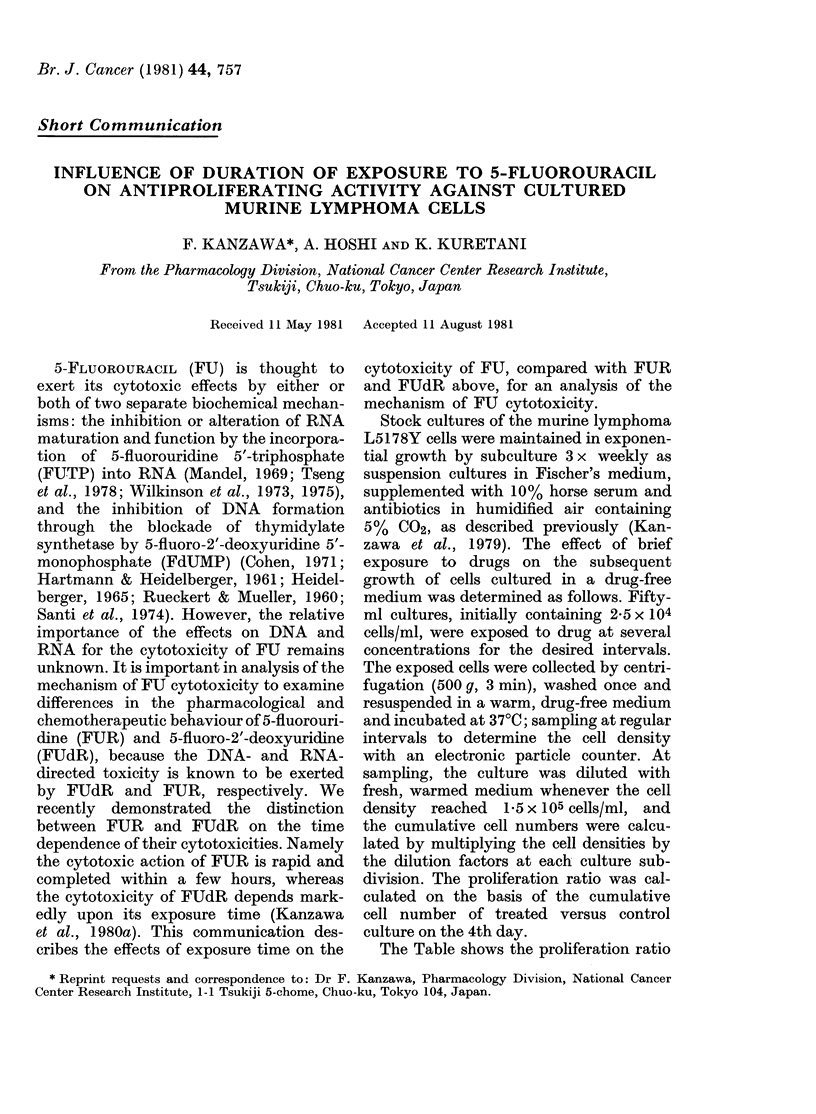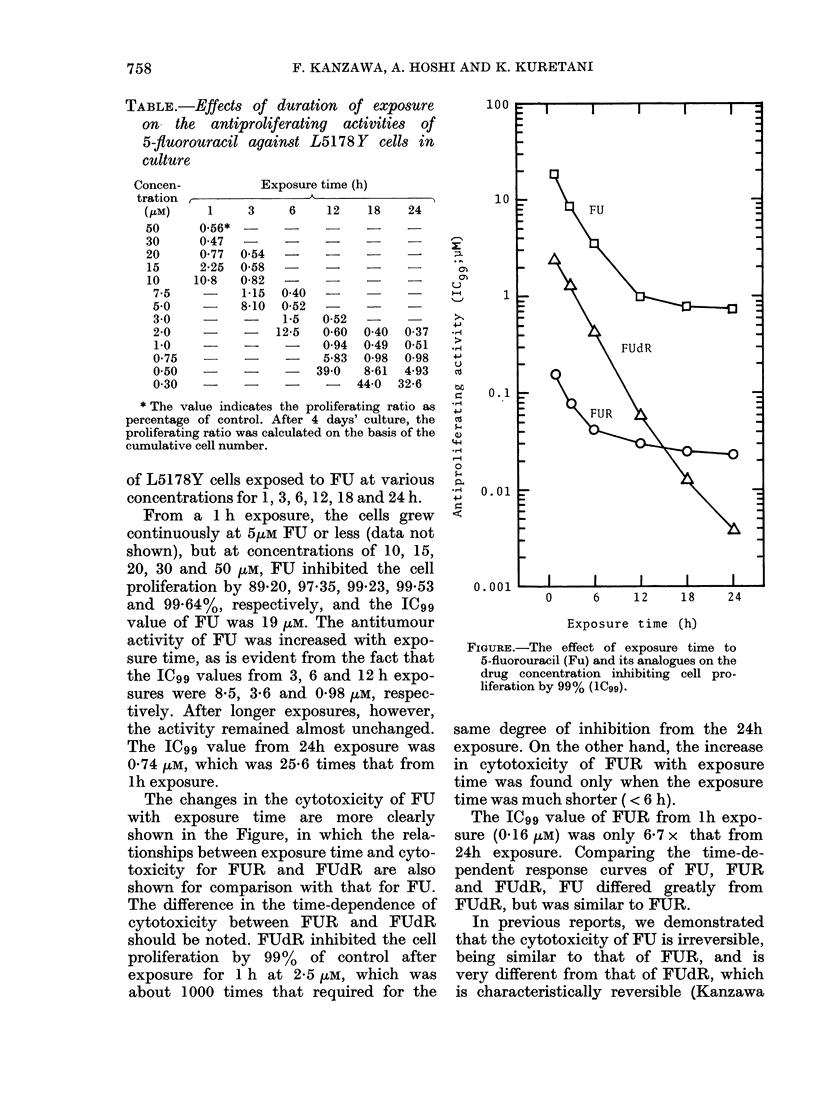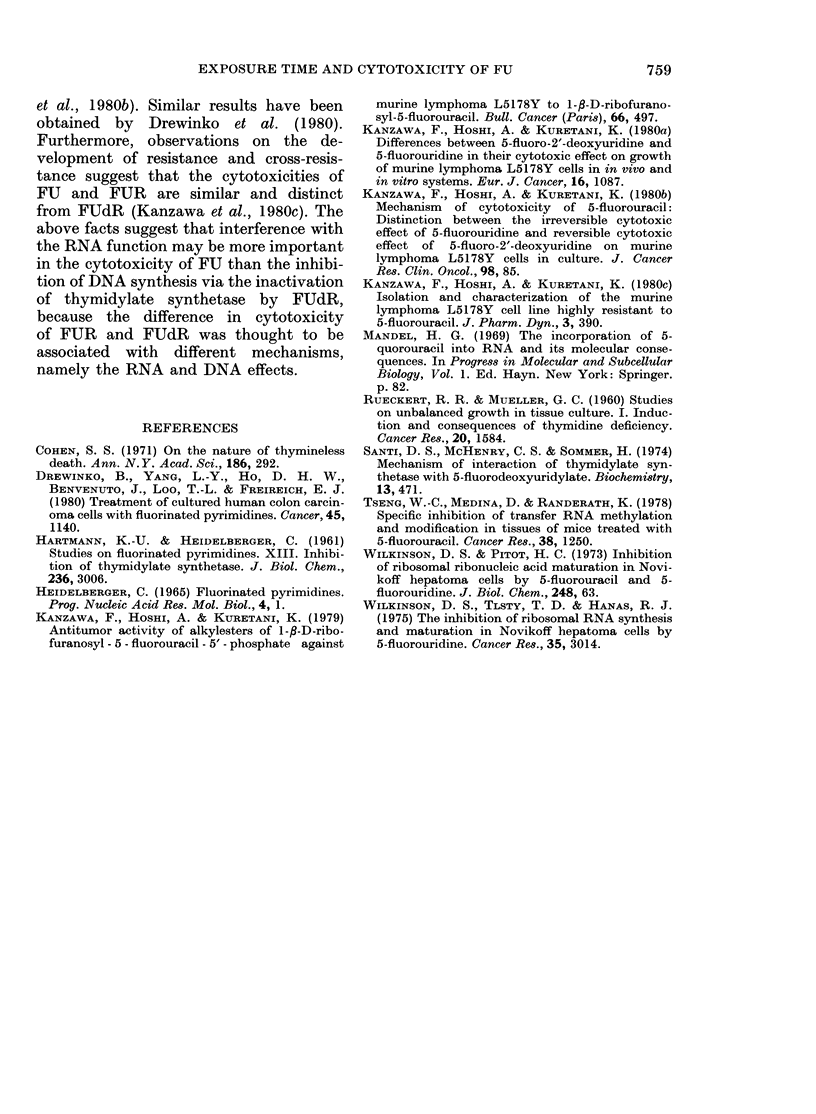# Influence of duration of exposure to 5-fluorouracil on antiproliferating activity against cultured murine lymphoma cells.

**DOI:** 10.1038/bjc.1981.265

**Published:** 1981-11

**Authors:** F. Kanzawa, A. Hoshi, K. Kuretani


*Br. J. Cancer* (1981) **44**, 757

***Short Communication***

### INFLUENCE OF DURATION OF EXPOSURE TO 5-FLUOROURACIL ON ANTIPROLIFERATING ACTIVITY AGAINST CULTURED MURINE LYMPHOMA CELLS

F. KANZAWA*, A. HOSHI AND K. KURETANI

*From the Pharmacology Division, National Cancer Center Research Institute, Tsukiji, Chuo-ku, Tokyo, Japan*

Received 11 May 1981 Accepted 11 August 1981

5-FLUOROURACIL (FU) is thought to exert its cytotoxic effects by either or both of two separate biochemical mechanisms: the inhibition or alteration of RNA maturation and function by the incorporation of 5-fluorouridine 5′-triphosphate (FUTP) into RNA (Mandel, 1969; Tseng *et al.*, 1978; Wilkinson *et al.*, 1973, 1975), and the inhibition of DNA formation through the blockade of thymidylate synthetase by 5-fluoro-2′-deoxyuridine 5′-monophosphate (FdUMP) (Cohen, 1971; Hartmann & Heidelberger, 1961; Heidelberger, 1965; Rueckert & Mueller, 1960; Santi *et al.*, 1974). However, the relative importance of the effects on DNA and RNA for the cytotoxicity of FU remains unknown. It is important in analysis of the mechanism of FU cytotoxicity to examine differences in the pharmacological and chemotherapeutic behaviour of 5-fluorouridine (FUR) and 5-fluoro-2′-deoxyuridine (FUdR), because the DNA- and RNA-directed toxicity is known to be exerted by FUdR and FUR, respectively. We recently demonstrated the distinction between FUR and FUdR on the time dependence of their cytotoxicities. Namely the cytotoxic action of FUR is rapid and completed within a few hours, whereas the cytotoxicity of FUdR depends markedly upon its exposure time (Kanzawa *et al.*, 1980*a*). This communication describes the effects of exposure time on the cytotoxicity of FU, compared with FUR and FUdR above, for an analysis of the mechanism of FU cytotoxicity.

Stock cultures of the murine lymphoma L5178Y cells were maintained in exponential growth by subculture 3× weekly as suspension cultures in Fischer's medium, supplemented with 10% horse serum and antibiotics in humidified air containing 5% $CO_2$, as described previously (Kanzawa *et al.*, 1979). The effect of brief exposure to drugs on the subsequent growth of cells cultured in a drug-free medium was determined as follows. Fifty-ml cultures, initially containing $2{\cdot}5 \times 10^4$ cells/ml, were exposed to drug at several concentrations for the desired intervals. The exposed cells were collected by centrifugation (500 *g*, 3 min), washed once and resuspended in a warm, drug-free medium and incubated at 37°C; sampling at regular intervals to determine the cell density with an electronic particle counter. At sampling, the culture was diluted with fresh, warmed medium whenever the cell density reached $1{\cdot}5 \times 10^5$ cells/ml, and the cumulative cell numbers were calculated by multiplying the cell densities by the dilution factors at each culture subdivision. The proliferation ratio was calculated on the basis of the cumulative cell number of treated versus control culture on the 4th day.

The Table shows the proliferation ratio

* Reprint requests and correspondence to: Dr F. Kanzawa, Pharmacology Division, National Cancer Center Research Institute, 1-1 Tsukiji 5-chome, Chuo-ku, Tokyo 104, Japan.

TABLE.—*Effects of duration of exposure on the antiproliferating activities of 5-fluorouracil against L5178Y cells in culture*

| Concentration (μM) | Exposure time (h) 1 | 3 | 6 | 12 | 18 | 24 |
|---|---|---|---|---|---|---|
| 50 | 0·56* | — | — | — | — | — |
| 30 | 0·47 | — | — | — | — | — |
| 20 | 0·77 | 0·54 | — | — | — | — |
| 15 | 2·25 | 0·58 | — | — | — | — |
| 10 | 10·8 | 0·82 | — | — | — | — |
| 7·5 | — | 1·15 | 0·40 | — | — | — |
| 5·0 | — | 8·10 | 0·52 | — | — | — |
| 3·0 | — | — | 1·5 | 0·52 | — | — |
| 2·0 | — | — | 12·5 | 0·60 | 0·40 | 0·37 |
| 1·0 | — | — | — | 0·94 | 0·49 | 0·51 |
| 0·75 | — | — | — | 5·83 | 0·98 | 0·98 |
| 0·50 | — | — | — | 39·0 | 8·61 | 4·93 |
| 0·30 | — | — | — | — | 44·0 | 32·6 |

* The value indicates the proliferating ratio as percentage of control. After 4 days' culture, the proliferating ratio was calculated on the basis of the cumulative cell number.

of L5178Y cells exposed to FU at various concentrations for 1, 3, 6, 12, 18 and 24 h.

From a 1 h exposure, the cells grew continuously at 5μM FU or less (data not shown), but at concentrations of 10, 15, 20, 30 and 50 μM, FU inhibited the cell proliferation by 89·20, 97·35, 99·23, 99·53 and 99·64%, respectively, and the $IC_{99}$ value of FU was 19 μM. The antitumour activity of FU was increased with exposure time, as is evident from the fact that the $IC_{99}$ values from 3, 6 and 12 h exposures were 8·5, 3·6 and 0·98 μM, respectively. After longer exposures, however, the activity remained almost unchanged. The $IC_{99}$ value from 24h exposure was 0·74 μM, which was 25·6 times that from 1h exposure.

The changes in the cytotoxicity of FU with exposure time are more clearly shown in the Figure, in which the relationships between exposure time and cytotoxicity for FUR and FUdR are also shown for comparison with that for FU. The difference in the time-dependence of cytotoxicity between FUR and FUdR should be noted. FUdR inhibited the cell proliferation by 99% of control after exposure for 1 h at 2·5 μM, which was about 1000 times that required for the

FIGURE.—The effect of exposure time to 5-fluorouracil (Fu) and its analogues on the drug concentration inhibiting cell proliferation by 99% ($IC_{99}$).

same degree of inhibition from the 24h exposure. On the other hand, the increase in cytotoxicity of FUR with exposure time was found only when the exposure time was much shorter (< 6 h).

The $IC_{99}$ value of FUR from 1h exposure (0·16 μM) was only 6·7 × that from 24h exposure. Comparing the time-dependent response curves of FU, FUR and FUdR, FU differed greatly from FUdR, but was similar to FUR.

In previous reports, we demonstrated that the cytotoxicity of FU is irreversible, being similar to that of FUR, and is very different from that of FUdR, which is characteristically reversible (Kanzawa

*et al.*, 1980*b*). Similar results have been obtained by Drewinko *et al.* (1980). Furthermore, observations on the development of resistance and cross-resistance suggest that the cytotoxicities of FU and FUR are similar and distinct from FUdR (Kanzawa *et al.*, 1980*c*). The above facts suggest that interference with the RNA function may be more important in the cytotoxicity of FU than the inhibition of DNA synthesis via the inactivation of thymidylate synthetase by FUdR, because the difference in cytotoxicity of FUR and FUdR was thought to be associated with different mechanisms, namely the RNA and DNA effects.

### REFERENCES

Cohen, S. S. (1971) On the nature of thymineless death. *Ann. N.Y. Acad. Sci.*, **186**, 292.

Drewinko, B., Yang, L.-Y., Ho, D. H. W., Benvenuto, J., Loo, T.-L. & Freireich, E. J. (1980) Treatment of cultured human colon carcinoma cells with fluorinated pyrimidines. *Cancer*, **45**, 1140.

Hartmann, K.-U. & Heidelberger, C. (1961) Studies on fluorinated pyrimidines. XIII. Inhibition of thymidylate synthetase. *J. Biol. Chem.*, **236**, 3006.

Heidelberger, C. (1965) Fluorinated pyrimidines. *Prog. Nucleic Acid Res. Mol. Biol.*, **4**, 1.

Kanzawa, F., Hoshi, A. & Kuretani, K. (1979) Antitumor activity of alkylesters of 1-$\beta$-D-ribofuranosyl-5-fluorouracil-5′-phosphate against murine lymphoma L5178Y to 1-$\beta$-D-ribofuranosyl-5-fluorouracil. *Bull. Cancer (Paris)*, **66**, 497.

Kanzawa, F., Hoshi, A. & Kuretani, K. (1980*a*) Differences between 5-fluoro-2′-deoxyuridine and 5-fluorouridine in their cytotoxic effect on growth of murine lymphoma L5178Y cells in *in vivo* and *in vitro* systems. *Eur. J. Cancer*, **16**, 1087.

Kanzawa, F., Hoshi, A. & Kuretani, K. (1980*b*) Mechanism of cytotoxicity of 5-fluorouracil: Distinction between the irreversible cytotoxic effect of 5-fluorouridine and reversible cytotoxic effect of 5-fluoro-2′-deoxyuridine on murine lymphoma L5178Y cells in culture. *J. Cancer Res. Clin. Oncol.*, **98**, 85.

Kanzawa, F., Hoshi, A. & Kuretani, K. (1980*c*) Isolation and characterization of the murine lymphoma L5178Y cell line highly resistant to 5-fluorouracil. *J. Pharm. Dyn.*, **3**, 390.

Mandel, H. G. (1969) The incorporation of 5-quorouracil into RNA and its molecular consequences. In *Progress in Molecular and Subcellular Biology, Vol.* 1. Ed. Hayn. New York: Springer. p. 82.

Rueckert, R. R. & Mueller, G. C. (1960) Studies on unbalanced growth in tissue culture. I. Induction and consequences of thymidine deficiency. *Cancer Res.*, **20**, 1584.

Santi, D. S., McHenry, C. S. & Sommer, H. (1974) Mechanism of interaction of thymidylate synthetase with 5-fluorodeoxyuridylate. *Biochemistry*, **13**, 471.

Tseng, W.-C., Medina, D. & Randerath, K. (1978) Specific inhibition of transfer RNA methylation and modification in tissues of mice treated with 5-fluorouracil. *Cancer Res.*, **38**, 1250.

Wilkinson, D. S. & Pitot, H. C. (1973) Inhibition of ribosomal ribonucleic acid maturation in Novikoff hepatoma cells by 5-fluorouracil and 5-fluorouridine. *J. Biol. Chem.*, **248**, 63.

Wilkinson, D. S., Tlsty, T. D. & Hanas, R. J. (1975) The inhibition of ribosomal RNA synthesis and maturation in Novikoff hepatoma cells by 5-fluorouridine. *Cancer Res.*, **35**, 3014.